# European Academy of Nursing Science and the Swedish Society of Nursing Summer Conference 2017: The Future Direction of European Nursing and Nursing Research: Malmö, Sweden. 04-05 July 2017

**DOI:** 10.1186/s12912-017-0218-2

**Published:** 2017-06-27

**Authors:** Denise F. Polit, Souraya Sidani, David A. Richards, Ania Willman, Alison Kitson, Marleen Huijben-Schoenmakers, Arno Rademaker, Erik Scherder, Kaisa Bjuresäter, Maria Larsson, Ulrika Bergsten, Margaret Coulter Smith, Claire Pearson, Savina Tropea, Fiona O’May, Lindesay Irvine, Robert Rush, Rowena Wilson, Anne C. Rahn, Anja Behncke, Anke Buhl, Sascha Köpke, Maria Goreti Da Rocha Rodrigues, Maya Shaha, Markus Hjelm, Doris M. Bohman, Ania Willman, Jimmie Kristensson, Göran Holst, Anne Øverlie, Mariska Machiels, Sandra M. G. Zwakhalen, Silke F. Metzelthin, Jan P. H. Hamers, Laura Darcy, Katarina Karlsson, Kathleen Galvin, Ann Van Hecke, Simon Malfait, Kristof Eeckloo, Anna Råberus, Inger K. Holmström, Annelie J. Sundler, Ate Dijkstra, Berit Gesar, Carina Bååth, Hanne Hedin, Ami Hommel, Nancy Helou, Maya Shaha, Anne Zanchi, Cecilie Varsi, Elin Børøsund, Jelena Mirkovic, Elizabeth Marcheschi, Lena Von Koch, Hélène Pessah-Rasmussen, Marie Elf, Åsa Audulv, Åsa Kneck, Andrea Koppitz, Susanne de Wolf-Linder, Geneviève Blanc, Georg Bosshard, Thomas Volken

**Affiliations:** 1 Humanalysis, Inc., Saratoga Springs, NY USA; 2 Griffith University, Centre for Health Practice Innovation, Brisbane, QLD Australia; 3 School of Nursing, Ryerson University, Toronto, Canada; 4 University of Exeter Medical School, Exeter, UK; 5 Malmö University, Malmö, Sweden; 6 Adelaide Nursing School, Faculty of Health and Medical Sciences, University of Adelaide, Adelaide, Australia; 7 Department of Clinical Neuropsychology, Free University of Amsterdam, Amsterdam, The Netherlands; 8 Institute of Health Sciences, Karlstad University, Karlstad, Sweden; 9 Rheumatology Department, Sahlgrenska University Hospital, Gothenburg, Sweden; 10 Division of Nursing, School of Health Sciences, Queen Margaret University, Edinburgh, UK; 11 Institute of Neuroimmunology and Multiple Sclerosis (INIMS), University Medical Centre Hamburg-Eppendorf, Hamburg, Germany; 12 MIN Faculty, Unit of Health Sciences and Education, University of Hamburg, Hamburg, Germany; 13 Institute of Social Medicine and Epidemiology, Nursing Research Unit, University of Lübeck, Lübeck, Germany; 14 Workers Welfare Association Schleswig-Holstein, Kiel, Germany; 15 HES-SO University of Applied Sciences and Arts Western Switzerland, School of Health Sciences, Geneva, Switzerland; 16 Institute of Higher Education and Research in Healthcare, University of Lausanne and University Hospital of Lausanne, Lausanne, Switzerland; 17 Department of Health, Blekinge Institute of Technology, SE-371 79 Karlskrona, Sweden; 18 Department of Health Sciences, Lund University, SE-221 00 Lund, Sweden; 19 Department of Care Science, Malmö University, SE-211 18, Malmö, Sweden; 20 Department of Master- and post graduate education, Diakonova University College, Oslo, Norway; 21 Department of Health Services Research, CAPHRI School for Public Health and Primary Care, Maastricht University, Maastricht, Netherlands; 22 Faculty of Caring Science, Work Life and Social Welfare, University of Borås, Borås, Sweden; 23 School of Health Sciences, University of Brighton, Brighton, UK; 24 Department of Public Health, University Centre for Nursing and Midwifery, Faculty of Medicine and Health SciencesGhent University, 9000 Ghent, Belgium; 25 Ghent University Hospital, 9000 Ghent, Belgium; 26 University of Borås, Borås, Sweden; 27 Mälardalen University, Västerås, Sweden; 28 Uppsala University, Uppsala, Sweden; 29 Department of Healthcare & Welfare, NHL University of Applied Sciences, Leeuwarden, The Netherlands; 30 Department of Clinical Sciences Lund, Orthopaedics, Lund University, Lund, Sweden; 31 Faculty of Health, Sciences, and Technology, Department of Health Sciences, Karlstad University, Karlstad, Sweden; 32 County Council of Värmland, Karlstad, Sweden; 33 Department of Orthopaedic, Falun Hospital, Falun, Sweden; 34 Department of Care Science, Malmö University, Malmö, Sweden; 35 Department of Health Care Science, Lund University, Lund, Sweden; 36 Department of Orthopaedic, Skåne University Hospital Lund, Lund, Sweden; 37 University of Health Sciences (HESAV), University of Applied Sciences of Western Switzerland (HES-SO), 1011 Lausanne, Switzerland; 38 University of Lausanne-Faculty of Biology and Medicine, University Institute of Higher Education and Research in Healthcare (IUFRS), Lausanne, Switzerland; 39 Department of Internal Medicine, Services of Nephrology, Diabetes and Endocrinology, Centre Hospitalier Universitaire Vaudois (CHUV), Lausanne, Switzerland; 40 Medical Department, Center for Shared Decision Making and Collaborative Care Research, Oslo University Hospital, Oslo, Norway; 41 Chalmers University, Gothenburg, SE-412 96 Sweden; 42 Departmentt of Neurobiology, Care Sciences and Society, Karolinska Institutet, Stockholm, SE-171 77 Sweden; 43 Faculty of Medicine, Lund University, Lund, SE-221 00 Sweden; 44 School of Education, Health and Social Studies, Dalarna University, Falun, SE-791 88 Sweden; 45 Department of Nursing Science, Mid Sweden University, Sundsvall, Sweden; 46 Department of Health Care Sciences, Ersta Sköndal Univeriversity College, Stockholm, Sweden; 47 Department of Health, Institute of Nursing, Research, and Development, Zurich University of Applied Sciences, CH-8401 Winterthur, Switzerland; 48 Clinic for Geriatric Medicine, University Hospital of Zurich, CH-8091 Zurich, Switzerland; 49 Department of Health, Institute of Public Health, Zurich University of Applied Sciences, CH-8401 Winterthur, Switzerland

## KEYNOTE PRESENTATIONS

### K1 My life in research methods in nursing

#### Denise F. Polit^1,2^ (denisefpolit@gmail.com)

##### ^1^ Humanalysis, Inc., Saratoga Springs, NY, USA; ^2^Griffith University, Centre for Health Practice Innovation, Brisbane, QLD, Australia

Dramatic changes have occurred during my 40+ years of teaching research methods to nurses and nursing students, including important transformations within the profession, an explosive growth in published research by nurses, greater inter-professional collaboration, increased funding opportunities, and stunning methodologic and technologic advances. In this talk I will describe some of the major ways in which nursing research has evolved over the past 4 decades by tracing content revisions in my textbook *Nursing Research: Generating and Assessing Evidence for Nursing Practice—*now in its 10th edition and still the world’s #1 textbook on research methods for nurses. The earliest editions of this book had no mention of such key concepts as *evidence-based practice, mixed-methods research, complex interventions,* and *systematic reviews;* now, entire chapters are devoted to these topics. Indeed, it was not until the 3rd edition of the book (1987) that qualitative research was given serious coverage, and now many chapters of the book describe methods for conducting high-quality qualitative inquiry. My presentation will focus especially on the key revisions I introduced in the 10th edition, which was published in 2016 (2017 copyright date). In particular, I will discuss a topic about which I have become passionate—the measurement and interpretation of *clinical significance* (distinct from statistical significance) in nursing studies. I will also describe revisions that I am planning to make in the 11th edition, which will be published in early 2020. In all editions published thus far, I have strived to provide guidance on how to do research with rigor and integrity—that is, how to “get it right” when answering a research question. In the 11th edition I plan to devote considerably more attention to what nurse researchers can do to “get it right” in asking their research questions. In this regard, I will provide an overview of strategies for improving the *relevance* and *applicability* of nursing research.

### K2 Complex Interventions: areas for further development

#### Souraya Sidani (ssidani@ryerson.ca)

##### School of Nursing, Ryerson University, Toronto, Canada

**Background**

Complex interventions consist of multiple components addressing different behaviors at the individual, organization or community levels. Whereas advances have been made in their design and evaluation, additional work is needed to enhance their relevance and transferability.

**Purpose**

This methodological presentation offers a glimpse at additional strategies that can be incorporated in early phases of developing and evaluating complex interventions; the goal is to have efficient interventions that incorporate acceptable, feasible and most effective components.

**Strategies and Implications**

In the development and modeling phase, conceptual analysis of the linkages among the context, the problem, the intervention’s components and the outcomes is foundational for clearly identifying the active ingredients and the most appropriate approach for operationalizing them. This analytic work can be translated into a logic model, which is a tool to assist with the presentation of the complex intervention to stakeholder groups and to inform the evaluation study. Partnering with stakeholders in this analysis and formally assessing their acceptability of each intervention component (using quantitative, qualitative or combined methods) are important strategies to improve the relevance and hence initiation, implementation and effectiveness of complex interventions. In the pilot evaluation phase, quantitative and qualitative methods are applied to examine: 1) the feasibility and satisfaction with each component, the intervention as-a-whole, and mode and dose of delivery; 2) the mechanisms underlying the effects of the intervention and its components on intended and unintended outcomes; and 3) the contextual factors (related to the characteristics of the target population, professionals, setting) that facilitate and/or hinder the fidelity of intervention and components implementation, satisfaction, and outcomes. The results of such pilot evaluation generate preliminary yet comprehensive understanding of what are the active ingredients of the intervention, which components delivered in what format, mode and dose are important to produce beneficial outcomes, for whom and under what conditions. This knowledge is critical for refining the complex intervention prior to evaluating its effectiveness and for determining its transferability to different contexts.

### K3 Humanitus: returning to the essential principles of clinical nursing care

#### David A. Richards^1^, Ania Willman^2^

##### ^1^ University of Exeter Medical School, Exeter, United Kingdom; ^2^ Malmö University, Malmö, Sweden

###### **Correspondence:** David A. Richards (d.a.richards@exeter.ac.uk)

In a discussion of nursing’s legacy and how to take the discipline’s research forward, we will first highlight contemporary issues in the western world’s health care of today. Against the background of reduced staffing levels, fewer hospital beds and shorter lengths of stay in hospitals, and the fact that nurses, as frontline staff, are under great pressure, we will question nursing’s role in addressing human needs. We will also ask the question: ‘how do nurses know how to care?’

Our points of departure will be Meleis’ (2012) [1] characteristics that define the nursing perspective as: “*the nature of caring science as a human science, the practice aspects of nursing, the caring relationships that nurses and patients develop and the health and wellness perspective*.” Additionally, we will critically examine caring as a concept using the writings of Rodgers (2000) [2]: “*the understanding and the use of concepts is dependent on the context where it occurs*”. We will ask if caring, and consequently nursing, is the same today as it was in Florence Nightingale’s time and how we understand nursing today.

We will then go onto questions of how a nurse should nurse. If nursing is truly a science, how are we to operationalise the collection, analysis and contribution of data to that science? There are many worries. For example, has the rush to ‘prove’ that nurses can take on the roles of medics and others left essential nursing care – helping the person who “*lacks knowledge, physical strength, or the will to act for himself as he would ordinarily act in health*” (Henderson, 1966) [3] – bereft of evidence for true nursing action. Whilst we might not all agree with Nightingale [4,5] that, “*To understand God's thoughts we must study statistics, for these are the measure of His purpose*”, could a more mature attitude from nursing science to statistical analyses not lead to more certainty of nursing action? Finally, to paraphrase Nightingale herself, has the science of nursing led to “*feelings wasted in words”* and not “*distilled into actions which bring results*”?

**References**

1. Meleis A.I. (2012). Theoretical Nursing: Development and Progress. Lippincott: USA.

2. Rodgers B.L. (2000). Concept analysis: an evolutionary view. In: Concept Development in Nursing: Foundations, Techniques and Applications. 2nd edn. (Rodgers B.L & Knafl K.A, eds.) Saunders, Philadelphia, pp 77-102.

3. Henderson, V. (1966). The Nature of Nursing. Macmillan, New York.

4. Nightingale, F. Letter to a friend, quoted in *The Life of Florence Nightingale* (1913). Cook, ET. p. 94

5. Nightingale, F. as quoted in ‘Chance Rules: An Informal Guide to Probability, Risk, and Statistics’ (1999) Everitt, B. p. 137

### K4 What is Person-Centred Fundamental Care and how do we do it?

#### Alison Kitson (alison.kitson@adelaide.edu.au)

##### Adelaide Nursing School, Faculty of Health and Medical Sciences, University of Adelaide, Adelaide, Australia

**Background**

Despite the significant investment in time and resources in strengthening the academic base of nursing, there still exists a lack of agreement about the nature of basic or fundamental nursing care; how it is delivered; and what the role of the qualified nurse is in that activity. This is a global phenomenon.

**Materials and Methods**

Inductively derived statements from expert nurses from a variety of clinical, cultural and academic settings were used to generate a conceptual framework (called the Fundamentals of Care Framework) [1].

**Results**

Person-Centered Fundamental Care (PCFC) (the goal) is achieved by (the nurse) utilizing the Practice Process (PP) [2] which is shaped by three major dimensions: the nature of the relationship between the nurse and the patient (R) [3,4]; how the fundamentals of care are integrated across the patient’s physical, psychosocial and relational needs and their position on the dependence-independence continuum (I-FOC) [5,6]; and how the context or environment where care happens is conducive to optimize outcomes (C). The resultant theoretical formula can be presented as PCFC = PP (R + I-FOC + C) [7].

**Conclusion**

This theory informing point-of-care interaction between the nurse and the patient around fundamental care has growing face validity. It requires further testing in multiple practice settings before its effectiveness in improving fundamental care can be claimed.

**Acknowledgements**

I should also like to acknowledge colleagues at the Adelaide Nursing School (Rebecca Feo, Tiff Conroy, Jan Alderman, Rick Wiechula, & Philippa Rasmussen) who have worked tirelessly with me on developing and refining the concepts that have shaped the Fundamentals of Care Framework and also members of the International Learning Collaborative (ILC) who have also contributed significantly to the ideas around fundamental care, in particular to Yvonne Wengstrom, Jack Needleman, Asa Muntlin-Athlin, Eva Jangland and Erik Soerensen.

**References**

1. Kitson A, Conroy T, Kuluski K, Locock L, Lyons R (2013) *Reclaiming and redefining the Fundamentals of Care: Nursing’s response to meeting patients’ basic human needs.* Synthesis of work from 2012 ILC Workshop. (Original Research Report) School of Nursing, University of Adelaide ISBN 978-0-9872126-2-7

2. Feo, R., Conroy, T., Alderman, J., & Kitson A. (2017) Implementing patient-centred fundamental care in practice. *Nursing Standard,* 31, 32, 52-61.

3. Wiechula R, Conroy T, Kitson AL, Marshall RJ, Whitaker N, Rasmussen P. (2016) Umbrella review of the evidence: What factors influence the caring relationship between a nurse and a patient? *Journal of Advanced Nursing.* 72 (4), 723-734.

4. Feo, R., Conroy, T., Marshall, R.J., Rasmussen, P., Wiechula, R., and Kitson, A.L., (2016) Using holistic interpretative synthesis to create practice-relevant guidance for person-centred fundamental care delivered by nurses. Nursing Inquiry DOI: 101111/min.12152

5. Kitson, A.L., Dow, C., Calabrese, J.D., Locock, L., and Athlin, A.M., (2013) Stroke survivors' experiences of the fundamentals of care: A qualitative analysis*. Int J Nurs Study.*50, 392-403.

6. Kitson AL. & Muntlin Athlin, A. (2013) Development and preliminary testing of a framework to evaluate patients’ experiences of the fundamentals of care: a secondary analysis of three stroke survivor narratives. *Nurs Res Pract*, ID572437

7. Kitson AL, Towards a Theory of Person-Centered Fundamental Care: the case for re-examining theories informing point-of-care interaction between nurse and patient. (under review) *Nursing Research Special Edition on Theories i*

## SPEAKER PRESENTATIONS

### Nursing interventions (S1-S7)

#### S1 Can practice undertaken by patients be increased simply through implementing agreed national guidelines? An observational study

##### Marleen Huijben-Schoenmakers, Arno Rademaker, Erik Scherder

###### Department of Clinical Neuropsychology, Free University of Amsterdam, Amsterdam, The Netherlands

####### **Correspondence:** Marleen Huijben-Schoenmakers (marleenhuijben@gmail.com)

This abstract is not included here as it has already been published [1].

**Reference**

[1] Huijben-Schoenmakers M, Rademaker A, Scherder E. ‘Can practice undertaken by patients be increased simply through implementing agreed national guidelines?’ An observational study. Clinical Rehabilitation 2013;27(6):513-520.

#### **S2** Patients’ views of quality of care related to different models of contact nurse in Swedish cancer care

##### Kaisa Bjuresäter, Maria Larsson

###### Institute of Health Sciences, Karlstad University, Karlstad, Sweden

####### **Correspondence:** Kaisa Bjuresäter (kaisa.bjuresater@kau.se)

**Background**

The increasing number of patients treated for and surviving cancer has drawn attention to the need for current systems of care to change in order to meet patients’ requirements of supportive care. In order to increase patient participation and reduce variations of quality of care, the Swedish government stated in 2011 that all cancer patients should be assigned to a Contact Nurse (CN) and receive an individual care plan, but different models for organizing CN care have been identified. The aim of this study was to describe patients’ perceptions of quality of care received by CN related to different models of CN care.

**Material and Methods**

The study was carried out in 2015 in seven County Councils coordinated by one Regional Cancer Centre in Sweden. Three models for CN were previously identified with various care intensity and structured follow-up care. Data collection was carried out by means of a modified Quality from Patients’ Perspective (QPP) with additional items regarding CN scope of practice. Each item was evaluated by perceived reality (PR) and subjective importance (SR). Responses ranged from 1(totally disagree) to 4(totally agree). Data was collected twice (four and eight months after start of treatment) from patients (n = 107) with cancer of the head and neck, colo-rectal, breast and prostate. Descriptive and non-parametric statistics were used.

**Results**

Results for the total group showed that patients got information about treatment (m = 3.1), but received information regarding side-effects (m = 2.4) and self-care to a lesser extent (m = 2.4). Patients also reported that supportive care regarding symptoms was low (m = 2.2) and the possibility to participate in rehabilitation (m = 2.0). There were differences between the three models regarding received information (ns), support from the CN (p = 0.032) and opportunities to participate in own care (p = 0.011). The proportion of patients who had received a written care plan varied between the models (model 1: 50%, model 2: 43%, model 3: 25%) as well as proportions of patients who had discussed their rehabilitation with their CN (44/19/18%).

**Conclusion**

These findings have identified important areas for improvement in the provision of a comprehensive and high quality cancer care carried out by a CN. There are different models regarding CN care which implies that the goal of an equal cancer care is not accomplished.

#### S3 The Gothenburg Nurse-led Study – a randomized study evaluating the efficacy of nurse-led clinic based on person-centred care compared to regular care in rheumatology outpatient clinic

##### Ulrika Bergsten (ulrika.bergsten@vgregion.se)

###### Rheumatology department, Sahlgrenska University Hospital, Gothenburg, Sweden

**Background**

Rheumatoid arthritis (RA) is a chronic joint disease. Common symptoms are pain, swollen joints, stiffness, fatigue and disabilities in everyday life. The treatment of the disease is focused on minimizing the inflammation and new medicines are developed and have a great effect for some patients. Based on “regular care” data from 2012, only 30% of out-patient care of patients with RA (at the local hospital) reached the low disease activity at follow-up. Knowledge from nursing research shows that the patients need more than medicine to cope with this disease. There is need to have good skills in self-care and knowledge of the disease and treatment to manage the symptoms. The present study aimed to compare this “regular care” with a model based on nurse-led clinics with person-centred care and tight disease control.

**Purpose**

To compare a nurse-led clinic including person-centred care and tight control with “care as usual” in patients with rheumatoid arthritis (RA) and moderate/high disease activity.

**Project description**

Study population: Patients with RA, 18-80 years old, with moderate/high Disease Activity Score of 28 joints (DAS28 > 3.8) and disease duration > 2 years in a 6-month randomized controlled study with a 6-month open follow-up. Intervention group (N = 36): Nurse-led visits every 6 weeks, with structured person-centred care and evaluation of disease activity. If disease remission was not reached, pharmacological treatment was modified according to a predefined algorithm. A health plan was created to specify the goals from the patients with focus on how to improve health. The control group (N = 34) was treated according to “care as usual” with visits to physician every 6-12 months.

**Outcome measures**

Primary outcome measure is change in Diseases Activity Score (DAS) at week 26. Secondary outcomes are; quality of life, self-efficacy, disability, emotional well-being, pain, fatigue, sleep and satisfaction at week 26 and 50. The study finished in February 2017.

**Preliminary results**

The first results from week 26 (all patients have reached week 26) are under process and there are differences in favour for the interventions group within disease activity score and in other scores. The last visits in the study were due in February 2017 so the results will be available before the conference.

**Conclusions**

If this study shows that nurse-led clinic is effective due to medical goal and caring aspects, this model could easily be implemented in the health care system.

Trial reg: Clin gov: NCT02019901

#### S4 Exploring nursing perspectives on moving and handling in older people with osteoporosis in acute care settings

##### Margaret Coulter Smith, Claire Pearson, Savina Tropea, Fiona O’May, Lindesay Irvine, Robert Rush, Rowena Wilson

###### Division of Nursing, School of Health Sciences, Queen Margaret University, Edinburgh, United Kingdom

####### **Correspondence:** Margaret Coulter Smith (msmith1@qmu.ac.uk)

**Background**

Osteoporosis is highly prevalent worldwide and is associated with increased risk of low trauma fracture (LTF) [1], increased morbidity and mortality [2]. Major advances in diagnosis, management and prevention of secondary fractures have occurred [3] but implications for acute nursing care are less well documented. This project investigated practitioners’ experiences of caring for people with osteoporosis, knowledge of the disease, explored implications for moving and handling, reported patients’ care experiences, and developed education for frontline staff.

**Materials and methods**

An exploratory multiple methods design comprised qualitative research with healthcare professionals and people (aged 60+) with confirmed osteoporosis, an education feasibility and evaluation study with NHS staff, development of a complex education intervention, dissemination, and project evaluation. An interactive website [4] www.lydiaosteoporosis.com introduced an online complex education intervention using an Instructional Design model [5].

**Data Collection**

Qualitative interviews were conducted with purposive samples of healthcare professionals (N = 32) and patients with confirmed osteoporosis aged 60+ years (N = 16). A pre/post test observational study evaluated an education workshop intervention with a convenience sample (N = 51) of healthcare staff. A larger complex education intervention was designed. Stakeholder focus groups (including patients) informed development of an interactive website. Evaluation and dissemination phases included a stakeholder conference (and website launch), administration of online surveys, and seeking participants’ views on various dissemination activities [6].

**Findings**

Awareness of osteoporosis, increased fracture risk, and understanding of implications for care varied across staff. Fractures if sustained in hospital were reported to be mainly related to falls. Generic manual handling guidelines highlighted organisational, risk management and procedural aspects rather than healthcare agency (staff capacities and translation of guidelines into actions for individuals) or patient perspectives. Practitioners’ conceptions of moving and handling were influenced by discipline. Nursing’s unique contribution in moving and handling was under stated by staff. Patients reported mainly positive experiences but one sustained fractures during moving and handling for an investigation. Overall the project was positively evaluated.

**Discussion**

Moving and handling can be viewed as a complex intervention [7]. Nurses’ unique contribution to patient care within moving and handling requires greater exploration.

**Conclusions**

Nursing’s contribution and patient experiences of moving and handling are important areas for investigation. Collaborative action research and evaluation are in progress to further disseminate findings and online education.

**Acknowledgements**

Grateful thanks to the patients and staff who participated in this project, to Queen Margaret University for supporting the project, and the private anonymous benefactor who funded the work.

**References**

1. SIGN 2015 Management of osteoporosis and the prevention of fragility fractures Guideline No.142, ISBN 978 1 909103 35 1, Published March 2015, Scottish Intercollegiate Guidelines Network, Health Improvement Scotland http://sign.ac.uk/guidelines/fulltext/142/index.html Accessed 29.09.2016

2. IOF 2015 International Osteoporosis Foundation. Osteoporosis Facts and Statistics. http://www.iofbonehealth.org/facts-statistics Accessed 27th September 2016

3. Kanis J A, McCloskey E V, Johansson H, Cooper C, Rizzoli R, Reginster J Y. European guidance for the diagnosis and management of osteoporosis in post menopausal women. 2013 Osteoporos Int 24: 23–57. doi 10.1007/s00198-012-2074-y

4. Lydia Osteoporosis Project Website http://www.lydiaosteoporosis.com Accessed 22nd September 2016

5. van Merrienboër J J G, Kirschner P A. Ten Steps to Complex Learning. A Systematic Approach to Four-component Instructional Design. 2013. 2nd edition, New York, Routledge

6. Coulter Smith M, Pearson C, O’May F, Tropea S, Irvine L, Rush R, Wilson R. Final Project Report for the Lydia Osteoporosis Project. 2016a Queen Margaret University, Edinburgh http://eresearch.qmu.ac.uk/4419/1/4419.pdf Accessed 29.09.2016

7. Coulter Smith M, O’May F, Tropea S, Berg J. Framing moving and handling as a complex healthcare intervention within the acute care of older people with osteoporosis: A qualitative study Journal of Clinical Nursing 2016b; 25: 2906- 2920. doi:10.1111/jocn.13344 http://onlinelibrary.wiley.com/doi/10.1111/jocn.13344/epdf Accessed 29.09.2016

#### S5 Implementation of a guideline-based programme on physical restraint reduction in home care and nursing homes in northern Germany – multi-centre before-after study

##### Anne C. Rahn^1,2^, Anja Behncke^3^, Anke Buhl^4^, Sascha Köpke^3^

###### ^1^ Institute of Neuroimmunology and Multiple Sclerosis (INIMS),University Medical Centre Hamburg-Eppendorf, Hamburg, Germany; ^2^ MIN Faculty, Unit of Health Sciences and Education, University of Hamburg, Hamburg, Germany; ^3^ Institute of Social Medicine and Epidemiology, Nursing Research Unit, University of Lübeck, Lübeck, Germany; ^4^Workers Welfare Association Schleswig-Holstein, Kiel, Germany

####### **Correspondence:** Anne C. Rahn (a.rahn@uke.de)

**Background**

Physical restraints such as bedrails and belts are used regularly in care facilities and home care, despite a lack of evidence for their effectiveness in preventing falls and injuries in care recipients.

We aimed to implement a guideline-based multicomponent programme on physical restraints reduction that has been shown to be effective in nursing home care.

**Material and Methods**

Before-after study conducted in home care services and nursing homes of the “Workers Welfare Association” in northern Germany. Intervention materials, originally developed for nursing homes, were adapted for use in home care. A total of four one-day training courses for nurses were conducted. Participants received the guideline on physical restraints, associated materials (e.g. leaflet and brochures) and structured telephone counselling for 12 months. Prior to the intervention and after one year, prevalence of physical restraint use was assessed by direct observation. The primary endpoint was the percentage of care recipients with at least one physical restraint after one year.

**Results**

Nine home care facilities and 15 nursing homes took part in the study and 34 nurses were trained. In nursing homes, the number of residents with physical restraints was reduced from 13.4% (15 facilities, 103/771 residents) to 6.8% (13 facilities, 49/719 residents), ARR 6.6% (p = 0,001).

In home care, the number of clients with physical restraints was reduced from 5.3% (7 services, 38/719 clients) to 4.3% (8 services, 26/605 clients), ARR 1% (p = 0.4).

**Conclusions**

The guideline-based programme could successfully be adapted and implemented for the needs of home care services and nursing homes of the “Workers Welfare Association”, a large care provider in Germany. However, not all invited services agreed to take part in the intervention. Regular training and updates are necessary for permanent implementation. The limited data on home care suggest that the programme could also be effective here, warranting evaluation by a randomised controlled trial.

#### S6 Review: a life review intervention grounded in a nursing framework

##### Maria Goreti Da Rocha Rodrigues^1,2^, Maya Shaha^2^

###### ^1^HES-SO University of Applied Sciences and Arts Western Switzerland, School of Health Sciences, Geneva, Switzerland; ^2^Institute of Higher Education and Research in Healthcare, University of Lausanne and University Hospital of Lausanne, Lausanne, Switzerland

####### **Correspondence:** Maria Goreti Da Rocha Rodrigues (Gora.darocha@hesge.ch)

**Background**

Patients facing advanced cancer experience high levels of existential distress due to being confronted with their own mortality, which leads to feelings of lack of sense, or discouragement [1]. Interventions relieving existential distress to promote patients’ dignity are important. Dignity at the end of life and existential suffering constitute a major concern of nurses. Interventions based on a suitable theoretical framework need to be developed. Therefore, Shaha’s middle range theory [2] has been linked with Newman’s grand theory [3]. The emerging framework provides a basis for the development of a life-review intervention, called Revie⊕. This framework supports the conceptualization and implementation of a feasibility study, which aimed at exploring the feasibility of Revie⊕, which comprises a positive, patient-centered approach. In addition, acceptability by the patients and the nurses who conducted the intervention was determined.

**Materials and methods**

For linking the two nursing theories with different abstraction levels “integrative theorizing” as proposed by Kolcaba and Kolcaba [4] is employed. Heuristic criteria are employed to determine the compatibility: Shared assumptions, cultural applicability, disciplinary boundaries, nursing education, focus of care, process or product distinction, shared values, scientific orientation. A Venn diagram is used to illustrate the intersection of the two theories and to obtain new insights. Based on the newly created framework a life-review intervention, entitled Revie⊕, was developed.

A mixed methods design was used for this feasibility study, which was conducted at a Swiss university hospital. A total of 41 patients with advanced cancer participated. A total of eight nurses conducted the intervention. Patients’ and nurses’ views and perspectives were obtained with semi-structured interviews, one focus group and questionnaires.

**Results**

The results demonstrate good acceptability of Revie⊕ for both patients and the nurses conducting the intervention. Revie⊕ is considered beneficial by nurses for their professional posture and the patients. This intervention is oriented on a positive approach, valuing resources and centered on the patients’ values and preferences.

**Conclusion**

To develop a life review intervention for patients with advanced cancer, a suitable framework was created by linking one grand nursing theory and one middle-range nursing theory. To our knowledge, this is the first feasibility study of a life review intervention based on a framework with a nursing perspective that focuses on personal growth and on changes relating to the experience of cancer.

Trial registration ISRCTN12497093.

**References**

1. Kissane DW. The relief of existential suffering. Arch Intern Med. 2012;172(19):1501-5.

2. Shaha M. The Omnipresence of cancer [Cumulative thesis in partial fulfilment]. Witten, Herdecke: Department of Nursing Science; 2014.

3. Newman MA. Health as expanding consciousness. 2 ed. USA: Jones and Bartlett Publishers; 1994.

4. Kolcaba K, Kolcaba R. Integrative Theorizing: Linking Middle Range Nursing Theories with the Neuman System Model. In: Neuman B, Fawcett J, editors. The Neuman System Model. 5th ed: Pearson Education; 2011.

#### S7 Health and social care staff members’ experiences of a case management intervention focusing on improving continuity of care for older persons with complex health needs - a qualitative analysis

##### Markus Hjelm^1,2^, Doris M. Bohman^1^, Ania Willman^1,3^, Jimmie Kristensson^2^, Göran Holst^1,2^

###### ^1^Department of Health, Blekinge Institute of Technology, SE-371 79 Karlskrona, Sweden; ^2^Department of Health Sciences, Lund University, SE-221 00 Lund, Sweden; ^3^Department of Care Science, Malmö University, SE-211 18 Malmö, Sweden

####### **Correspondence:** Markus Hjelm (markus.hjelm@bth.se)

**Background**

Complex health systems make it difficult to ensure a continuity of care for older persons with multi-morbidity and risk leading to a fragmented care for this group [1-3]. Fragmented care could affect the quality and safety of the care provided [4]. Case management has been suggested as an intervention that could address health systems’ fragmented care of older persons with complex health needs [1,2]. To better understand and to advance the progress of case management interventions, there is a need for knowledge from perspectives possibly influencing the intervention. One such perspective is the organisational perspective e.g., the staff members. As of today, there is a need to extend knowledge regarding case management as experienced by staff working within the involved parts of the health system. Such knowledge could allow for us to better design for these aspects in upcoming case management interventions targeting older persons with complex health needs. Thus, the aim of this study was to explore health and social care staff members’ experiences with a case management intervention focusing on improving continuity of care for older persons, 65 and over, with complex health needs.

**Methods and materials**

The study design was a qualitative interview study. Data was collected through individual interviews with ten health and social care staff members, including managers, all conducted in 2013. Interview data was subjected to thematic analysis utilising an inductive approach.

**Results**

The data revealed three themes illustrating staff members’ experiences with the intervention: 1) could bridge gaps in an insufficient health system; 2) emerging improvements call for engagement and 3) an intervention in the mist with vague goals and elements.

**Conclusions**

The findings indicate that the staff members acknowledged the case management intervention’s ability to improve continuity of care for older persons with complex health needs. The intervention's purpose was perceived to be vague, indicating a need for enhanced educational efforts regarding its content. The intervention was perceived to provide potential ways of working towards improving parts of the health system. This improvement depended on engagement amongst all involved parties.

**References**

1. Oeseburg B, Wynia K, Middel B, Reijneveld SA. Effects of case management for frail older people or those with chronic illness: a systematic review. Nurs Res. 2009 Jun;58(3):201–10.

2. Goodwin N. Reviewing the evidence on case management: lessons for successful implementation. Int J Integr Care. 2011 Oct;11:e146.

3. Fortin M, Soubhi H, Hudon C, Bayliss EA, van den Akker M. Multimorbidity’s many challenges. BMJ. 2007 May 19;334(7602):1016–7.

4. Shaw S, Rosen R, Rumbold B. What is integrated care. An overview of integrated care [Internet]. London: Nuffield Trust; 2011. [cited 2016 Oct 12]. Available from: http://www.nuffieldtrust.org.uk/sites/files/nuffield/publication/what_is_integrated_care_research_report_june11_0.pdf

### Humanitus (S8-S14)

#### S8 The partner's perspective on inflammatory bowel disease

##### Anne Øverlie (anne.overlie@diakonova.no)

###### Dept. of Master- and post graduate education, Diakonova University College, Oslo, Norway

Inflammatory Bowel Disease (IBD) is known to have great implications on the patient’s adjustment to the changes brought on by the disease. This chronic disease, however, also afflicts the partner by forcing him/her to make considerable adaptations in order to cope with the unforeseen lifestyle changes. Knowledge of the partners’ coping strategies when living with a sick partner with IBD is sparse. The aim of the study was to reveal and investigate the experiences of the partner living with a person with IBD.

Nine partners participated in a qualitative study based on a semi-structured interview guide. Informants were five women and four men with an age span from 29 to 71 years. The interviews were analyzed using thematic content analysis. Data collection took place during the sick partners’ hospitalization in the autumn of 2008.

Four themes were selected:

1. Partners expressed worry, anxiety, greater workload, and curtailment of shared experiences. The loss of spontaneity called for planning.

2. The loss of the other as (s)he had been. Witnessing the emotional strain of the ill person, their pain and distress were troublesome. Partners did not share thoughts and worries with friends and family.

3. Partners managed to create some time needed by themselves.

4. They expressed a need for individually tailored information, support, and follow-up from the nursing staff.

No gender differences were found in the partners’ experiences. However, the partners with long life span experience with IBD had accepted the consequences in contrast to those of short life span with the disease. They longed for a medical treatment of any kind.

The illness created considerable adjustments for the partner due to changes leading to the inability of the ill person to contribute on an equal footing. Nursing staff should be aware of the partners’ need for information and understanding to enhance their coping mechanisms. The findings are relevant to nursing staff with particular regard to their interaction with the healthy partners.

#### S9 Towards better communication in nursing homes between nurses and people with dementia: design of a communication intervention

##### Mariska Machiels, Sandra M. G. Zwakhalen, Silke F. Metzelthin, Jan P. H. Hamers

###### Department of Health Services Research, CAPHRI School for Public Health and Primary Care, Maastricht University, Maastricht, Netherlands

####### **Correspondence:** Mariska Machiels (m.machiels@maastrichtuniversity.nl)

**Background**

Although nurses often report communication difficulties in caring for people with dementia (PWD), evidence-based interventions to improve communication during daily nursing care are scarce [1]. Therefore, we developed a theory-informed intervention with the aim to improve communication between nurses and PWD.

**Materials and methods**

Development was done using the Behaviour Change Wheel [2]. First, ideal communication was defined (targeted behaviour) based on a systematic review [1], additional (scientific) literature, and consultations of experts (n = 7). Second, to understand their current behaviour and to identify facilitators and barriers for the targeted behaviour, two focus group meetings with relevant stakeholders (e.g. nurses) were organized. Furthermore, observations of nurses and PWD (n = 9) during daily nursing care were conducted in a nursing home. Third, intervention functions and content was discussed with the focus group in a third meeting.

**Results**

Reviewing the literature and consulting experts has shown that ideal communication should be person-centred. Furthermore, next to verbal communication, attention should be paid to non-verbal communication, including the use of pictograms, objects, and touch. Additionally, the environment has to be recognisable and comprehensible for PWD. Focus group meetings and observations have shown that behaviour of nurses is often characterised by a task-oriented instead of person-centred approach. Furthermore, non-verbal communication (e.g., eye contact) is insufficiently used. Identified facilitators and barriers for the ideal communication relate to nurses’ characteristics (e.g., knowledge, awareness, and skills), social influences (e.g. family expectations, and team functioning), and other environmental factors (e.g., resources, and time). Intervention elements that the focus group considered useful were 1) training of nursing staff in verbal and non-verbal communication skills, 2) making use of the life story of PWD, 3) coaching on the job, 4) a collaborative view and related goals among co-workers regarding communication, 5) observing co-workers during interactions with people with dementia, and reflecting on own behaviour.

**Conclusions**

As a result of the process a theory-informed intervention to improve communication has been developed. The systematic development of the intervention and its final version will be presented.

**References**

1. Machiels M, Zwakhalen SMG, Metzelthin SF, Hamers JPH. Improving communication with people with dementia during daily nursing care: a systematic review. In: Lambregts J, van Merwijk C, de Groot B, editors. 5th European Nursing Congress Rotterdam, the Netherlands: Journal of Advanced Nursing; 2016.

2. Michie S, van Stralen MM, West R. The behaviour change wheel: a new method for characterising and designing behaviour change interventions. Implementation Science. 2011;6(1):1.

#### S10 Humanising children’s suffering during medical procedures

##### Laura Darcy, Katarina Karlsson^1^, Kathleen Galvin^2^

###### ^1^Faculty of Caring Science, Work Life and Social Welfare, University of Borås, Borås, Sweden; ^2^School of Health Sciences, University of Brighton, Brighton, United Kingdom

####### **Correspondence:** Laura Darcy (laura.darcy@hb.se)

**Background**

The views of children have historically been seen as unimportant – they have been viewed as unintelligent and unable to tell of their experiences or participate in care, resulting in dehumanisation. Recent research has given young vulnerable children a voice and highlighted the importance of caring humanly for sick children [1,2]. A conceptual framework consisting of eight dimensions of humanisation has been proposed by Todres, Galvin and Holloway (2009) [3] which can highlight the need for young children to be cared for as human beings: Insiderness, Agency, Uniqueness, Togetherness, Meaning – making, Personal journey, Sense of Place and Embodiment. The aim of this study is to demonstrate the value of a humanising theoretical framework in paediatric care illustrated by examples of young children’s suffering when undergoing medical procedures.

**Materials and Methods**

In two separate Swedish studies 20 children (3-7 years of age) with a variety of diagnoses were interviewed about their experiences of everyday life with cancer or their experiences of undergoing painful medical procedures. Parents’ and nurses’ views were welcomed as complimentary to child data. Interviews had been analysed qualitatively by either content analyses or by phenomenological and life world herme-neutic approaches. In the present study, a secondary inductive qualitative content analysis of the results has been made based on the proposed dimensions of humanisation/dehumanisation.

**Results**

The eight dimensional framework Illustrated several forms of dehumanisation: Objectification –children’s opinions and experiences are seldom requested; Passivity – the use of restraint still happens and negatively affects the child; Homogenisation – children are viewed as their diagnosis; Isolation – children sense separation from parents, siblings and friends; Loss of meaning –appropriate information and preparation for the child is lacking; Loss of personal journey - everyday life functioning is affected making it difficult to see meaning; Dislocation – a sense of homelessness is experienced at home, at the hospital and at preschool/school; Reductionist view – medical procedures becomes the professional focus of care, not the child.

**Conclusion**

Dehumanisation occurs when humanising dimensions are obscured to a significant degree. Children’s own voices in care and research are required to correct the present power imbalance. Children require assistance in making sense of healthcare situations through play and preparation. Access to family and friends, being treated with dignity and encouragement to participate in care, will encourage humanising the dehumanisation illustrated in this study.

**References**

1. Darcy, L. (2015). The everyday life of young children through their cancer trajectory. Jönköping: School of Health Sciences.

2. Karlsson, K. (2015). “I’m afraid, I want my mommy”: Younger children’s, parents’ and nurses’ lived experiences of needle procedures in health care. Jönköping: Jönköping University, School of Health and Welfare.

3. Todres, L., Galvin, K. T., & Holloway, I. (2009). The humanization of healthcare: A value framework for qualitative research. International Journal of Qualitative Studies on Health and Well-being, 4(2), 68-77.

#### S11 Privacy or information during bedside shift report, who decides what matters at the point of care?

##### Ann Van Hecke^1,2^, Simon Malfait^1,2^, Kristof Eeckloo^1,2^

###### ^1^University Centre for Nursing and Midwifery, Faculty of Medicine and Health Sciences, Department of Public Health, Ghent University, 9000, Ghent, Belgium; ^2^Ghent University Hospital, 9000 Ghent, Belgium

####### **Correspondence:** Ann Van Hecke (Ann.VanHecke@UGent.be)

**Background**

Bedside shift reporting is commonly practised in healthcare. It is considered to enhance patient safety and encourage active patient involvement in healthcare decisions [1], and to improve communication [2]. Still, international studies have identified several barriers to engaging in such bedside shift reporting. Of particular interest is the fear of healthcare workers to infringe the patient’s privacy in semi-private rooms. This fear often leads to nurses being reluctant to use a shift-to-shift report at the bedside [2]. The aim of the presentation is to elaborate on differences between the perspective of nurses and patients related to privacy issues related to bedside shift reporting.

**Materials and methods**

In our study [3], nurses (n = 100) and patients (n = 40) were interviewed linked to 15 nursing wards (surgical, medical rehabilitation, geriatric, and stroke unit). To enhance trustworthiness, researcher triangulation was used.

**Preliminary results**

Nurses often reported the infringement of privacy as a barrier for using bedside shift reporting. Patients in a private room seemed to be more concerned with their privacy, and consider bedside shift reporting to be a method that could possibly infringe their privacy. This possible infringement is one of the reasons why they insist on having a private room. Patients in semi-private rooms have a different point of view. They indicate that in semi-private rooms the privacy is, mostly unaware, already infringed by healthcare workers. First, they indicate that other professions (e.g. physicians) often share information in semi-private rooms. Additionally, patients indicate that they are convinced that nurses will be cautious with sensitive information. Second, patients indicate that they often already share information with their neighbours. For patients, being informed and adequate information sharing seem to be more important.

**Conclusion**

The implementation of bedside shift reporting is often blocked by the nurses’ argument that this will infringe the patient’s privacy. Interviews with patients indicate that receiving information is perhaps more important than the possible infringement of privacy. This patient perspective could be essential to integrate in discussion related to interventions like bedside shift reporting.

**References**

1. Anderson CD, Mangino RR. Nurse shift report: who says you can’t talk in front of the patient? Nurs Adm Q. 2006;30(2):112–122.

2. Gregory S, Tan D, Tilrico M, Edwardson N, Gamm L. Bedside Shift Report: what does the evidence say? J Nurs Adm. 2014;44(10): 541–545.

3. Malfait S, Eeckloo K, Lust E, Van Biesen W, Van Hecke A. Feasibility, appropriateness, meaningfulness and effectiveness of patient participation at bedside shift reporting: mixed-method research protocol. J Adv Nurs. 2016;epub ahead of print.

#### S12 The nature of patient complaints: a resource for improvements of healthcare services?

##### Anna Råberus^1^, Inger K. Holmström^2,3^, Annelie J. Sundler^1^

###### ^1^University of Borås, Borås, Sweden; ^2^Mälardalen University, Västerås, Sweden; ^3^Uppsala University, Uppsala, Sweden

####### **Correspondence:** Annelie J. Sundler (annelie.sundler@hb.se)

**Background**

Patient complaints may be a valuable source for information required for good care. Even though the quality of healthcare is imperative, some patients get harmed rather than helped by healthcare services today. While quality improvements have been discussed, more knowledge is needed on how patient complaints can be used to improve patient safety and healthcare services.

**Aim**

To explore and describe patient complaints from the perspective of patients or relatives.

**Materials and methods**

A retrospective study with a descriptive design was conducted to explore patient complaints reported to a Patients’ advisory committee in Sweden. Data was collected in 2016 and handed out from the patient complaint reporting system. From a total sample of 5689 patients complaints reported in 2015 a selection of 170 complaints were included and analyzed in the study. The data was analysed with a qualitative method for conventional content analysis.

**Results**

Essential in reported complaints were complications or experiences of disadvantages and problems of healthcare and treatment. The reported complaints can be viewed from different levels; describing examples of individual health professionals failing in responsibility or treatment, or problems on an organizational level. These findings are illustrated through the description of seven themes describing complaints regarding; availability, communication, continuity and follow-up, patient harms, incidents and errors, attitudes, and healthcare without respect for the patient’s will.

**Conclusions**

Patient complaints described critical incidents and significant situations of importance for excellence in healthcare services. Experiences were described of patients or relatives suffering at the hands of healthcare professionals, even though it may not have been caused deliberately to them. This study will further discuss the need to acknowledge patient complaints for quality improvements and patient satisfaction of healthcare services.

#### S13 Using the Care Dependency Scale for identifying patients at risk for malnutrition

##### Ate Dijkstra (ate.dijkstra@nhl.nl)

###### Department of Healthcare & Welfare, NHL University of Applied Sciences, Leeuwarden, the Netherlands

**Background**

Older people are at risk of malnutrition [1]. One of the main reasons for malnutrition is insufficient energy intake and weight loss, which can lead to increased functional impairment and increased care dependency [2]. Nurses play a key role in the risk assessment of nutrition and information about each person’s food preferences can be found in the care plan. The aim of the study was to evaluate which items of the Care Dependency Scale (CDS) are significant for malnutrition for patients receiving home care or admitted to a residential or nursing home in the Netherlands.

**Materials and Methods**

The CDS is an instrument to be used in the first stage of the nursing process as a need and risk assessment tool [3]. The convenience sample consisted of 13019 participants, of whom 2422 received home care from 15 organisations, 4017 were patients from 67 residential homes, and 6580 were admitted in 105 nursing homes. Data were taken from the Dutch National Prevalence Survey of Care Problems that was carried out in April 2012 in Dutch healthcare settings. Outcomes were calculated using descriptive statistics, cross-tabulation, and logistic regression analysis.

**Results**

One-way Analysis of Variance (P < 0.05) outcomes showed a significant difference on each CDS item across the three healthcare settings. In home care, ‘Eating and Drinking’ and ‘Contact with others’ had the highest Odds Ratio of 1.8, suggesting a risk for malnutrition that is almost 2 times higher for clients who are dependent in this item. In residential and nursing homes, ‘Contact with others’ (Odds ratio of 1.8 respectively 2.0) indicates a 2 times higher risk for malnutrition. Results of the logistic regression analysis confirmed the above-mentioned findings.

**Conclusions**

Study outcomes showed that some CDS items indicate a potential risk for malnutrition. In practice, the use of the CDS for risk assessment of malnutrition must be combined with an individualized assessment based on nurses’ clinical experience and judgement.

**Acknowledgements**

We want to thank the Dutch National Prevalence Survey of Care Problems for providing their data.

**References**

1. Dijkstra A. Identifying residents at risk of care complications in care homes. Nurs Stand. 2016; 31(2):54-62.

2. Tannen A, Lohrmann C. Malnutrition in Austrian hospital patients. Prevalence, risk factors, nursing interventions, and quality indicators: a descriptive multicentre study. J Adv Nurs. 2012; 69(8):1840-1849.

3. Dijkstra A. Care Dependency: an assessment instrument for use in long-term care facilities. PhD thesis. Groningen: De Regenboog; 1998.

#### S14 Absence of fundamentals of nursing care and psychological support could be a reason for dependency four months after a hip fracture surgery

##### Berit Gesar^1^, Carina Bååth^2,3^, Hanne Hedin^4^, Ami Hommel^5,6,7^

###### ^1^Department of Clinical Sciences Lund, Orthopaedics, Lund University, Lund, Sweden; ^2^Faculty of Health, Sciences, and Technology, Department of Health Sciences, Karlstad University, Karlstad, Sweden; ^3^County Council of Värmland, Karlstad, Sweden; ^4^Department of Orthopaedic, Falun Hospital, Falun, Sweden; ^5^Department of Care Science, Malmö University, Malmö, Sweden; ^6^Department of Health Care Science, Lund University, Lund, Sweden; ^7^Department of Orthopaedic, Skåne University Hospital Lund, Lund, Sweden

####### **Correspondence:** Berit Gesar (berit.gesar@med.lu.se)

**Background**

The incidence of hip fractures is expected to increase due to the increase of the aging population. In Sweden, 40 percent of the patients are healthy and live an independent life before sustaining a hip fracture. The fracture results in declined function outcomes for almost 40 percent of these patients. An investigation of healthy patients’ perception of their own capacity to regain previous function at acute phase and follow-up interviews with the same persons four months later has not to our knowledge been conducted before.

**Aims and objectives**

To explore healthy older patients’ perception of their own capacity to regain pre-fracture function after a hip fracture, at the acute phase and follow up four months later.

**Methods**

The studies had an explorative inductive qualitative design. Qualitative semi-structured interviews were conducted (n = 30), two to five days after hip fracture surgery. Follow-up interviews were conducted (n = 25) in the patients’ home four months later. Data were analysed with inductive content analysis.

**Results**

Initially in the acute care setting patients believed in recovery and thought nothing would be altered. Since they adapted to the ward culture, patients became passive and became insecure about their future life situation. Patients’ influence on their recovery was limited [1]. Four months later the after effects from the hip fracture was seen as an interruption leading to lasting consequences for everyday life. Some participants had resigned, some strived in order to regain independency and some handled the situation by means of self-esteem, self-confidence and self-efficacy.

**Conclusion**

The professional purpose of recovery is to restore patients to their previous physical, mental and social capabilities after a hip fracture. Findings reveal that absence of psychological support may be one of the reasons for dependency four months after hip fracture surgery. Person-centred, holistic fundamental nursing care may have a mediating role in long-term functional outcomes for this group. Fundamentals of care could strengthen personal dignity, self-confidence, self-esteem, self-determination and perceived control. Meeting patients’ fundamental care needs are often poorly executed in acute care settings in Sweden.

**Acknowledgements**

We would like to thank all the study participants for their valuable contribution.

**References**

1. Gesar B, Hommel A, Hedin H, Bååth C. Older patients’ perception of their own capacity to regain pre-fracture function after hip fracture surgery- an explorative qualitative study. Int J Orthop Trauma Nurs. Forthcoming 2016. doi:10.1016/j.ijotn.2016.04.005

### Methodological Innovations in Nursing Research (S15-S19)

#### S15 Cross-Over trials’ use in nursing research: the nurse-led multidisciplinary self-care management program

##### Nancy Helou^1^, Maya Shaha^2^, Anne Zanchi^3^

###### ^1^University of Health Sciences (HESAV), University of Applied Sciences of Western Switzerland (HES-SO), 1011 Lausanne, Switzerland; ^2^University of Lausanne-Faculty of Biology and Medicine, University Institute of Higher Education and Research in Healthcare (IUFRS), Lausanne, Switzerland; ^3^Services of Nephrology, Diabetes and Endocrinology, Department of Internal Medicine, Centre Hospitalier Universitaire Vaudois (CHUV), Lausanne, Switzerland

####### **Correspondence:** Nancy Helou (nancy.helou@hesav.ch)

The cross-over design is rarely used in nursing clinical research. With the increase in chronic diseases and the need to develop nursing research in this field, cross-over may represent the ultimate randomized design that is able to overcome the heterogeneity of co-morbidities of different studies’ populations.

Cross-over designs allow efficient comparison of treatments when recruiting fewer participants and attaining the same level of statistical power as randomized controlled trials. It is for use more importantly in chronic diseases for comparison of participants’ responses to different treatments. Each participant receives treatment and serve of own control thus, overcoming the mixed effects related to heterogeneity of co-morbidities when comparing two different groups. The uniform strongly balanced design represents the ideal cross-over since it is able to overcome the statistical bias of carry-over effect. This design was applied in the randomized trial aiming to investigate the effect of a nurse-led multidisciplinary self-care management program (MSMP) on quality of life, self-care behavior, adherence to antihypertensive therapy, glycemic control and renal function of patients with diabetic kidney disease. Recent global health concern has been raised over diabetic kidney disease because it is a chronic disease associated with high morbidity, mortality and health care costs. Moreover, affected individuals present, in addition to diabetes and kidney disorders, different comorbidities such as arthritis, hypertension, and cardiovascular diseases, rendering the disease management more challenging.

The uniform balanced cross-over design was used with 32 participants randomized into four study arms. Each participant received twice, at different time intervals and over 12 months, three months of usual care alternating with three months of MSMP. Quality of life was evaluated using the Audit of Diabetes-Dependent Quality of Life scale, patient self-care behavior was measured using the Revised Summary of Diabetes Self-Care Activities, and adherence to anti-hypertensive treatment was assessed using the Medication Events Monitoring System. Blood glucose control was measured by glycated hemoglobin levels and renal function by serum creatinine, estimated glomerular filtration rate and urinary albumin/creatinine ratio. Analysis of the data considered estimate effect of each treatment based on participants’ treatment differences. Results showed improved general quality of life and self-care activities of diabetic kidney disease participants. Results of glycemic control and renal function were similar in the MSMP and the usual care groups.

#### S16 User participation for successful development of eHealth self-management interventions

##### Cecilie Varsi, Elin Børøsund, Jelena Mirkovic

###### Center for Shared Decision Making and Collaborative Care Research, Medical Department, Oslo University Hospital, Oslo, Norway

####### **Correspondence:** Cecilie Varsi (cecilie.varsi@rr-research.no)

**Background**

eHealth interventions have the potential to support patients in self-managing their illness, improve quality of life and enhance self-efficacy. Despite interest from patients and their health care providers, eHealth interventions are seldom used as intended and have high attrition rates. To avoid this, it is important to ensure that the interventions are designed in line with the users’ requirements and contexts. Therefore, at Center for Shared Decision Making and Collaborative Care Research, Oslo University Hospital, we use Service Design methods to involve end-users and other stakeholders in the whole process of design and development of eHealth interventions. This presentation will show how we integrate the Service Design methods into our work, by showing some examples.

**Materials and Methods**

Service Design (Stickdorn & Schneider 2011) is used as an approach to support the entire design and development process, aiming to elicit user wants and needs, and identifying how to best fit the eHealth intervention into the daily routines of patients and health care providers. Together with researchers and software developers, patients and health care providers are involved at each stage of intervention development. In the initial phase we use methods such as interviews and focus groups with stakeholders to identify their needs and requirements and observations to identify contextual conditions. The results are summarized into for example intervention Journey maps (“road maps”) or Personas (fictive patient characters). Based on gained insights, the next phases include series of workshops, where we together define opportunities, get inspiration, co-create ideas and develop intervention prototypes. Prototypes are then iteratively tested, validated and adapted through series of formative evaluations, using methods such as usability testing, think-aloud protocol, before the interventions are ready for final efficacy trials.

**Results**

We will present some examples of how Service Design methods can be used to involve different stakeholders into design and development of eHealth intervention. The close collaboration between researchers, software developers, patients and health care providers creates a genuine platform to develop solutions which are inspired by genuine behaviors, contexts and needs.

**Conclusions**

Even if the involvement of patients and health care providers is time-consuming and there is need for careful facilitation, the Service Design approach is useful for creating eHealth and other healthcare interventions that are in line with the users’ needs and requirements. In this way, we are able to create interventions that people want to use, and thus contribute to creating better health for people.

#### S17 Development of a methodology for the investigation of stroke patients’ rehabilitation in home settings

##### Elizabeth Marcheschi^1^, Lena Von Koch^2^, Hélène Pessah-Rasmussen^3^, Marie Elf^4^

###### ^1^ Chalmers University, Gothenburg, SE-412 96, Sweden; ^2^ Dept of Neurobiology, Care Sciences and Society, Karolinska Institutet, Stockholm, SE- 171 77 Sweden; ^3^ Faculty of Medicine, Lund University, Lund, SE-221 00, Sweden; ^4^ School of Education, Health and Social Studies, Dalarna University, Falun, SE-791 88, Sweden

####### **Correspondence:** Elizabeth Marcheschi (elizabeth.marcheschi@chalmers.se)

**Background**

A transformation in the way that healthcare systems and nursing services are delivered to patients can be seen at national and international levels. In Sweden, one of the major challenges regards the transfer and effectiveness of services outside specialized hospital units [1]. This is the case, for instance, of care and support provided to stroke patients, for whom rehabilitation activities are no longer performed within stroke units, but often occur in home settings [2]. The beneficial effects of home-based rehabilitation are mainly explained by investigations addressing the importance of patients’ centrality, social interactions, atmosphere, treatment planning and coordination [3]. The framework provided by the International Classification of Functioning suggests also the importance of considering the role played by the physical environment in supporting the individual functioning [4]. However, at present, a paucity of knowledge regarding what physical aspects of home settings facilitate vs hinder rehabilitation processes of stroke patients is found [5].The aim of the present work was thus, to reduce this knowledge gap by developing a method that could advance our understanding about the interaction between home settings and patients with stroke experience of it. The individual experience of the environment was conceptualized in line with knowledge from the field of environmental psychology, which entails information regarding emotional - cognitive responses and well-being related outcomes [6].

**Method**

A research protocol was developed based upon the joint effort and collaboration among experts from different disciplines; nursing research, medicine, architecture and environmental psychology. Several meetings were undertaken to discuss theories, concepts and methodologies connected to the topic of investigation with the purpose of merging together the different, yet complementary, knowledge held by these fields of study.

**Results**

A single approach, composed of mixed-methods and multi-perspective strategies, was developed. The former refers to the use of different tools (e.g. observational technique, behavioral mapping and interviews) to grasp the effect of the physical environment on users’ perception of social support, emotional - cognitive responses and on rehabilitation and well-being outcomes. The latter strategy entails the collection of information from different social actors (i.e nurses, patients, family members).

**Conclusion**

The proposed protocol adds knowledge to an almost unexplored area of research by assessing the role played by the physical environment in supporting stroke patients’ physical and psychosocial rehabilitation processes.

**References**

1. SOU 2016: 2. Effective care [in Swedish]. Retrieve from http://www.sou.gov.se/wp-content/uploads/2016/01/SOU-2016_2_Hela4.pdf.

2. Rousseaux, M., Daveluy, W., & Kozlowski, O. Value and efficacy of early supported discharge from stroke units. Annals of Physical and Rehabilitation Medicine. 2009; 52 (3) 224-233.

3. Meijer, R, & van Limbeek, J. Early supported discharge: a valuable alternative for some stroke patients. Lancet. 2005; 365 - 455-456.

4. WHO. International Classification of Functioning, Disability and Health. 2015; Available from: http://www.who.int/classifications/icf/icf_more/en/

5. Swedish Council on Health Technology Assessment. Early support discharge and continued rehabilitation in the home for elderly people after stroke: a systematic literature review [in Swedish]. 2015; Available from: http://www.sbu.se/sv/publikationer/SBU-utvarderar/tidig-koordinerad-utskrivning-och-fortsatt-rehabilitering-i-hemmiljo-for-aldre-efter-stroke/

6. Küller, R. Environmental assessment from a neuropsychological perspective. In: T. Gärling and G. W. Evans, (Eds.). Environment, Cognition, and Action. Oxford University Press, New York. 1991; pp 78-95.

#### S18 How to analyze time and change in qualitative longitudinal materials? Insights from a literature review of longitudinal qualitative studies in nursing

##### Åsa Audulv^1^, Åsa Kneck^2^

###### ^1^Department of Nursing Science, Mid Sweden University, Sundsvall, Sweden; ^2^Department of Health Care Sciences, Ersta Sköndal Univeriversity College, Stockholm, Sweden

####### **Correspondence:** Åsa Audulv (asa.audulv@miun.se)

**Background**

Longitudinal qualitative research can give new insights in social processes and experiences over time. In recent years, there has been a growing interest in conducting longitudinal qualitative research within nursing. However, the definition of what constitutes longitudinal qualitative research is unclear, the methodological literature scarce, and the variation of procedures great. This review of longitudinal qualitative articles within the nursing field aims to identify and describe various types of qualitative longitudinal approaches.

**Materials and Method**

Searches in pubmed identified over a hundred qualitative nursing articles with data collection over time. These articles were analyzed regarding 1) described analysis procedure, 2) how the results related to aspects of time and change, and 3) if results were person oriented vs category oriented.

**Results**

Five different types of longitudinal qualitative approaches were identified. In total, a large part of the papers described as having a longitudinal design performed a data collection over time, but did not integrate ideas of time or change in their analysis or results. Four fruitful approaches to analyzing longitudinal qualitative data were identified; time-line, pool, phase and pattern-oriented approaches. Articles classified as using any of these approaches have a clear perspective of time or change in the results. However, depending on type of approach different aspects of time, change, and process are in focus. Further, using different approaches yielded different kinds of results.

**Conclusion**

All approaches have pros and cons and researchers need to make informed decisions when choosing which approach they will take when analyzing qualitative longitudinal material.

#### S19 Pain intervention for people with dementia – what is essential in the development of a complex intervention

##### Andrea Koppitz^1^, Susanne de Wolf-Linder^1^, Geneviève Blanc^1^, Georg Bosshard^2^, Thomas Volken^3^

###### ^1^Department of Health, Institute of Nursing, Research, and Development, Zurich University of Applied Sciences, CH-8401 Winterthur, Switzerland; ^2^Clinic for Geriatric Medicine, University Hospital of Zurich, CH-8091 Zurich, Switzerland; ^3^Department of Health, Institute of Public Health, Zurich University of Applied Sciences, CH-8401 Winterthur, Switzerland

####### **Correspondence:** Andrea Koppitz (kopp@zhaw.ch)

**Background**

A large number of employees who care for people with dementia (PwD) in nursing homes lack expertise in the assessment of patients’ complex needs and often lack the necessary confidence to intervene accordingly (see abstract: Patient safety in nursing homes depends on effective communication; how are we doing?). The aim of this study is to develop a nurse-led intervention to build up nurses’ competencies in the care of PwD to prevent and to treat unstable health conditions.

**Material and Method**

This study is part of an ongoing nurse-led intervention trial in Switzerland (German Clinical Trials Register: DRKS00009726), where nurses with different skill mixes were individually trained to assess, intervene and evaluate common symptoms such as pain by an advanced nurse practitioner and a GP. The intervention was developed in line with the MRC framework and described on the basis of the TIDieR reporting guidelines. Nurses with different skill mix (n = 8: registered nurses, health care assistants, and auxiliary staff) evaluated the feasibility of the intervention and re-evaluated it after introduction and application attending two separate focus groups. Another two focus-groups with stakeholders (n = 8; health care professionals) were then asked about the usefulness of the intervention in the organisational context. Finally, nurses, who have received the intervention were then approached to participate either in an in-depth interview (n = 10) or in a focus group (n = 10) in order to evaluate the process and its intended outcome in line with the MORECare statement.

**Findings**

Principles, facilitators and barriers in planning, developing and implementing a complex nurse-led intervention in Swiss nursing homes were described. It became evident that individual (i.e palliative care experience) as well as organisational (i.e supporting structures) factors are important during the implementation of an intervention with a large training component. However, it also showed that nurses who felt supported, and felt that they worked in an appreciative and well-accepted environment, were more empowered to actively participate in the learning process.

**Conclusion**

Qualitative data to understand and address success or failure of such an intervention are important to improve the quality of a comprehensive geriatric assessment, followed by multiple interventions to address the complex symptom burden of PwD.

German Clinical Trials Register: DRKS00009726.

**Acknowledgement**

Swiss Federal Office of Public Health (BAG), Swiss Academy of Medical Sciences (SAMS), Swiss Alzheimer's Association, Ebnet Foundation, Switzerland

Publication charges for this supplement were funded by the conference.

